# Evaluation of reference genes for gene expression studies in mouse and N2a cell ischemic stroke models using quantitative real-time PCR

**DOI:** 10.1186/s12868-018-0403-6

**Published:** 2018-02-01

**Authors:** Yingbo Kang, Zhuomin Wu, De Cai, Binger Lu

**Affiliations:** Department of Pharmacy, The First Affiliated Hospital of Shantou University Medical College, Shantou, 515041 Guangdong China

**Keywords:** RT-qPCR, Reference genes, Ischemic stroke

## Abstract

**Background:**

Real-time reverse transcription quantitative polymerase chain reaction (RT-qPCR) is a critical tool for evaluating the levels of mRNA transcribed from genes. Reliable RT-qPCR results largely depend on normalization to suitable reference genes. Middle cerebral artery occlusion (MCAO) and oxygen–glucose deprivation/reoxygenation (OGD/R) are models that are commonly used to study ischemic stroke. However, the proper reference genes for RNA analysis in these two models have not yet been determined.

**Results:**

In this study, we evaluated the expression levels of six candidate housekeeping genes and selected the most suitable reference genes for RT-qPCR analyses of the cortices of MCAO mice and OGD/R-injured N2a cells. Four software programs, geNorm, NormFinder, BestKeeper and RefFinder, were used to validate the stabilities of the candidate reference genes. The results revealed that HPRT and 18S were the most stable reference genes in the cortices of MCAO mice and that β-actin and cyclophilin were the most stable reference genes in the OGD/R-injured N2a cells; in contrast, GAPDH and Sdha were the least stable genes in the cortices of MCAO mice and the OGD/R-injured N2a cells, respectively. Moreover, a combination of HPRT, 18S and cyclophilin was most suitable for normalization in analyses of the cortices of MCAO mice, and a combination of β-actin, cyclophilin, GAPDH, and 18S was most suitable for analyses of the OGD/R-injured N2a cells.

**Conclusions:**

This study provides appropriate reference genes for further RT-qPCR analyses of in vivo and in vitro ischemic stroke and demonstrates the necessity of validating reference genes for RNA analyses under variable conditions.

**Electronic supplementary material:**

The online version of this article (10.1186/s12868-018-0403-6) contains supplementary material, which is available to authorized users.

## Background

Real-time reverse transcription quantitative polymerase chain reaction (RT-qPCR) is one of the most important technologies applied to gene mRNA expression analysis in molecular biology. The greatest advantages of RT-qPCR are its high sensitivity and ease of operation [1]. However, some factors, such as the quality of the RNA, the efficiency of the reverse transcription, and the type of enzyme used for PCR amplification, as well as technical variations, can influence the reliability of PCR results [2].

To obtain reliable RT-qPCR results, one must use reference genes to normalize the data. Ideal reference genes should exhibit stable expression levels under different experimental conditions [3]. Housekeeping genes, which are usually expressed stably to maintain essential functions and include actin, tubulin, 18S ribosomal RNA and ubiquitin, are usually selected as reference genes. However, there is no universal reference gene that is suitable for every type of sample. A growing body of evidence indicates that almost every commonly used reference gene possesses different expression patterns in different cell types or under different conditions. The wrong choice of reference gene for the normalization of RT-qPCR data may give rise to wildly inaccurate results [4]. For example, hypoxanthine phosphoribosyltransferase (HPRT) was demonstrated to be a suitable reference gene in placental tissues from intrahepatic cholestasis of pregnancy [5] and in bladder cancer cells [6], while it was found to be unsuitable for articular cartilage [7]. GAPDH and β-actin, which are the most commonly used reference genes in molecular biological studies, have been demonstrated to be useful reference genes in human prostate cancer [8], while they have been found to be unsuitable for normalizing mRNA levels in human asthmatic airways [9]. Thus, screening for suitable reference genes for different conditions is a critical prerequisite of experimental design.

Stroke is a devastating disease that gives rise to high rates of mortality and adult disability worldwide. Ischemic stroke comprises approximately 87% of all stroke cases and has been the focus of numerous intensive studies [10]. The in vivo model of middle cerebral artery occlusion (MCAO) in C57BL/6 J mice and the in vitro model of oxygen-glucose deprivation/reoxygenation (OGD/R) in the mouse neuroblastoma cell line Neuro-2a (N2a) are widely used to investigate the delicate mechanism of stroke-induced neuronal death [11]. As one might expect, many studies have analyzed gene expression by RT-qPCR in these two model systems. However, not all reference genes for in vivo and in vitro ischemic injury assays have been properly validated.

In the present study, OGD/R-injured N2a cells and the ischemic cortices of C57 mice suffering MCAO were used as the experimental materials. Based on a survey of the literature, the expression levels of the six candidate reference genes that are most commonly used for in vivo and in vitro ischemic stroke models, including HPRT, β-actin, 18S, GAPDH, Sdha and cyclophilin, were evaluated by RT-qPCR. The expression stabilities of these genes were assayed using statistical programs, including geNorm [12], NormFinder [13], BestKeeper [14], the ΔCt method [15] and RefFinder. Additionally, the suitability of each chosen reference gene was then confirmed by RT-qPCR analysis.

## Results

### Specificity and efficiency of the primers

The expressional stabilities of the six candidate reference genes in both the MCAO mouse cortex and OGD/R-injured N2a cells were analyzed by RT-qPCR. These six candidate reference genes were selected from the literature because they are commonly used to normalize target gene expression in ischemic brains or OGD/R-injured N2a cells (Additional file 1: Table S1). For every candidate reference gene, the specificity of the primers was assessed by melting curve analysis and gel electrophoresis (2% agarose). As illustrated in Additional file 2: Fig. S1, the melting curves revealed a single peak and a single PCR product for each gene via gel electrophoresis, which demonstrated the good specificities of the primers used in the RT-qPCR analyses.

### Expression of reference genes in the MCAO mouse cortex and OGD/R-injured N2a cells

**Fig. 1:**
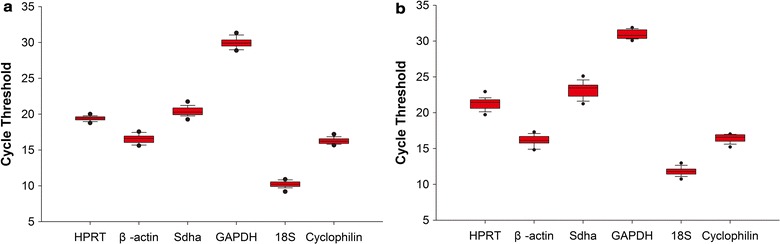
The range of Cp values of the candidate reference genes. **a** Boxplots of the Cp values of the candidate genes in the ischemic cerebral tissues from mice. **b** Boxplots of the Cp values of the candidate genes in the OGD/R-injured N2a cells. The dots indicate the 5th and 95th percentiles

First, mice were subjected to MCAO for various times (0.5, 1 and 1.5 h) followed by 24 h of reperfusion. The infarct volume was calculated using TTC-staining, and the results revealed that the intensity of the neuronal injury was increased during prolonged ischemia (Additional file 3: Fig. S2A and B). Moreover, the N2a cells were exposed to OGD for 3 h and then reoxygenated for 0, 4, 8, 12, 16 and 20 h. The effect of OGD/R on N2a cells viability was examined using CCK-8. The results revealed that injury was initially detected after 0 h of reoxygenation following 3 h of OGD and was continuously aggravated along with the extension of the reoxygenation (Additional file 3: Fig. S2C). Second, the expression levels of the six candidate reference genes in the cortices of the MCAO mice and in the OGD/R-injured N2a cells were evaluated by RT-qPCR using the Cp values of the samples. Descriptive statistics and Kolmogorov–Smirnov tests were used to evaluate the normalities of the Cp values for each gene involved in the cortices of the MCAO mice and in the OGD/R-injured N2a cells (Table 1). In both the MCAO mouse cortex and OGD/R-injured N2a cells, the expression of GAPDH exhibited the lowest abundance (the lowest median Cp value). Because the total RNA used for cDNA synthesis was consistent in all samples, and the threshold values for the six genes were the same, the Cp ranges of the candidate reference genes partly reflected the degree of variation. As illustrated in Fig. 1a, b, compared with the other candidate reference genes, greater variation in GAPDH gene expression and lesser variation in HPRT gene expression were found in the ischemic cortices of the mice. Furthermore, in the OGD/R-injured N2a cells, greater variation in Sdha gene expression and lesser variation in GAPDH were detected compared with the other candidates. The varied expressions of the six candidate reference genes indicated that these genes did not exhibit constant expression with the different degrees of ischemic damage in vivo or in vitro.

**Table 1:** Descriptive statistics regarding the reference gene Cp values in the tissues and cell samples

Gene	N	Mean	SD	Min Cp	Max Cp	KS-test *P*
Tissue						
HPRT	41	19.404	0.329	18.55	20.12	0.571
β-actin	41	16.551	0.570	15.61	17.67	0.542
Sdha	41	20.394	0.629	19.18	21.78	0.673
GAPDH	41	29.914	0.722	28.31	31.36	0.660
18S	41	10.212	0.457	9.15	11.13	0.458
Cyclophilin	41	16.274	0.419	15.55	17.26	0.736
Cells						
HPRT	23	21.313	0.803	19.62	23.15	0.610
β-actin	23	16.127	0.675	14.82	17.34	0.637
Sdha	23	23.14	1.07	21.18	25.23	0.670
GAPDH	23	30.89	0.572	30.03	31.91	0.843
18S	23	11.798	0.542	10.68	13.02	0.420
Cyclophilin	23	16.455	0.517	15.14	17.03	0.725

**Fig. 2:**
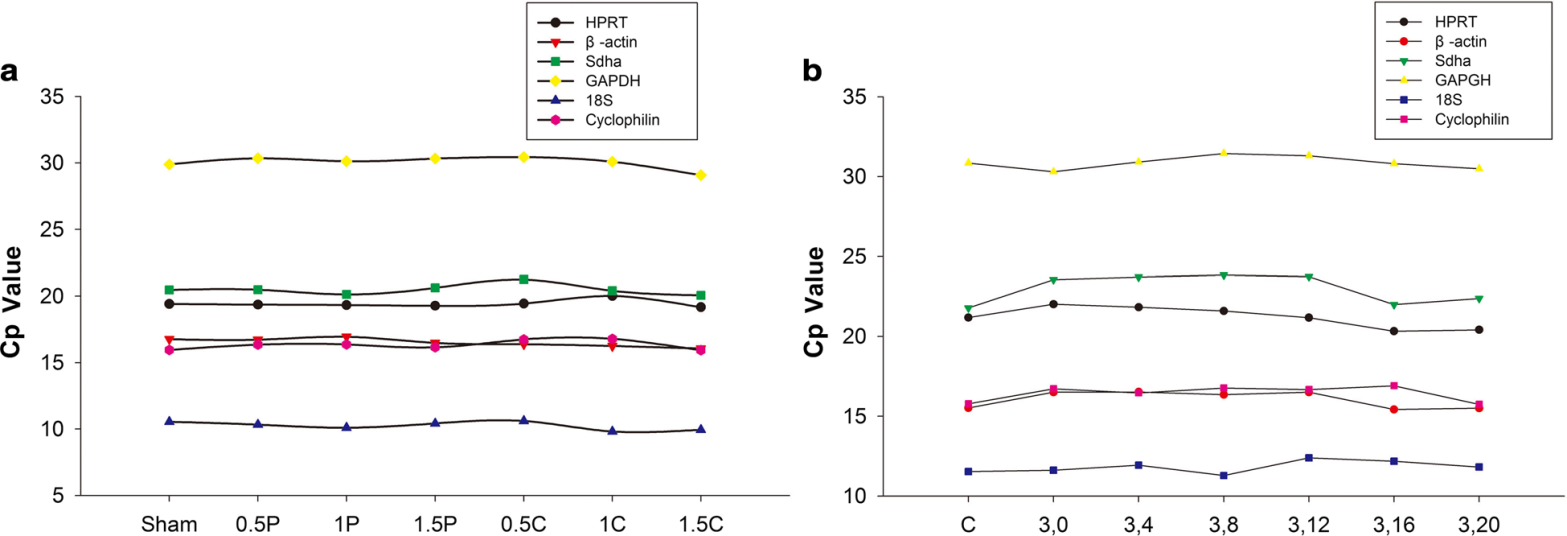
Variation of the reference gene expressions using the Cp values in the ischemic cerebral tissue of mice (**a**) and OGD/R-injured N2a cells (**b**)

Subsequently, the variations in gene expression were analyzed at different time points in reference to ischemic injury. The results demonstrated that the variations in the Cp values at the different time points in the cortices of the MCAO mice were comparable for all six candidate reference genes (Fig. 2a). However, a large variation in Sdha gene expression was found in the N2a cells with different degrees of OGD/R injury (Fig. 2b). An increased Cp value for the Sdha gene was initially detected after 0 h of reoxygenation, and this value continuously increased to its peak after 12 h of reoxygenation. Then, the Cp value of Sdha decreased to a nearly normal level after 16 h of reoxygenation.

### Expression stabilities of the candidate reference genes

**Fig. 3:**
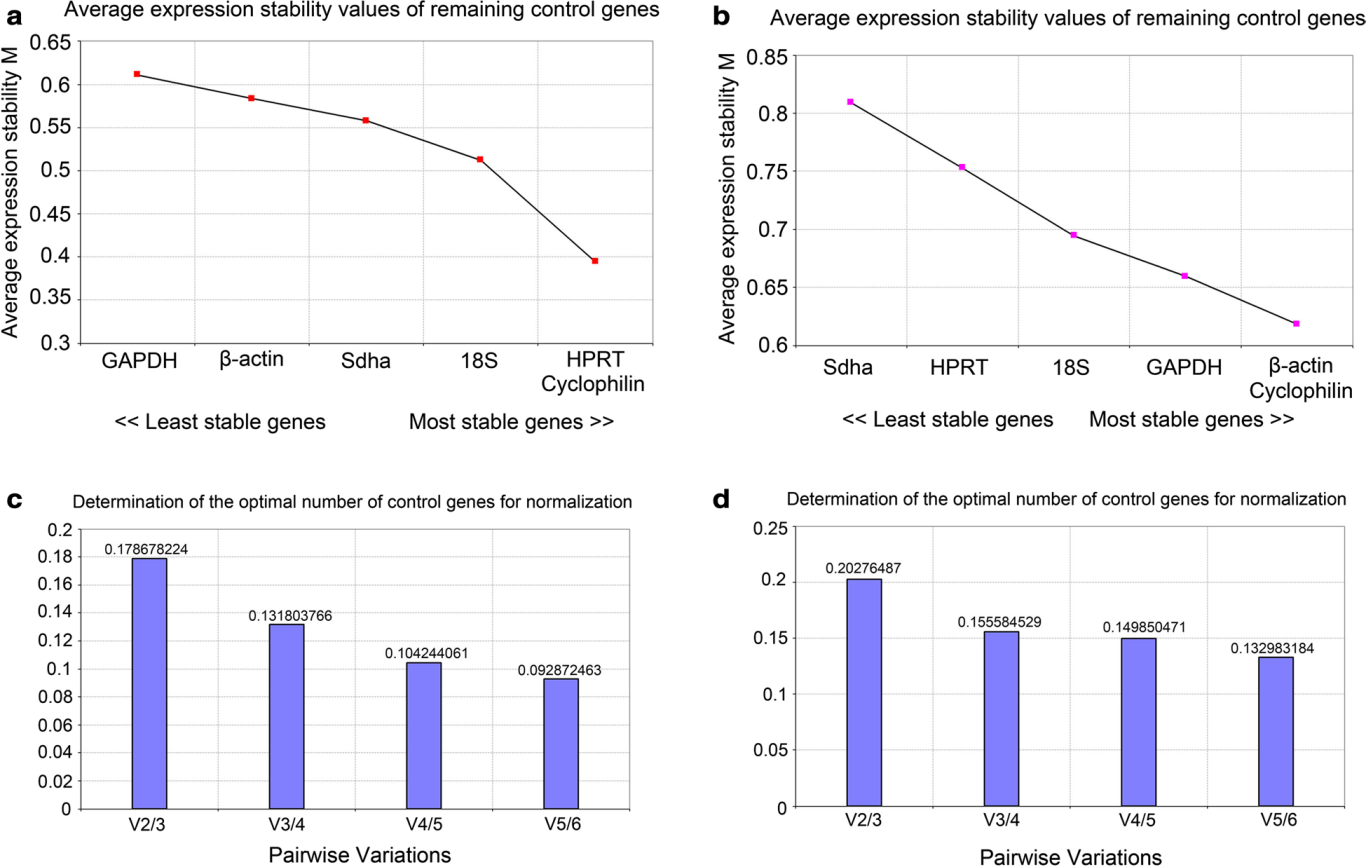
GeNorm analysis of the stabilities of the candidate reference genes. The average expression stabilities of the six candidate reference genes were analyzed in the ischemic cerebral tissue from mice (**a**) and in the OGD/R-injured N2a cells (**b**) by stepwise exclusion of the least stable genes. Higher M values represent lower stability. **c**, **d** Optimal numbers of reference genes required for normalization in ischemic cerebral tissue from mice (**c**) and OGD/R-injured N2a cells (**d**)

To estimate the variations in the expressions of the candidate reference genes, we began by applying the GeNorm analysis software. The principle of the gene-stability measure in GeNorm is that the expression ratio of two ideal reference genes is identical in all samples with different experimental conditions and cell types. Differences in the expression ratios of two candidate reference genes in different samples reflect the fact that one or both of the candidate genes are not constantly expressed. In this manner, the pairwise variation of every two genes is calculated, and the average pairwise variation of a particular gene with all other candidate genes is defined as the M value [12]. GeNorm analysis ranks gene stability using the M value, and a candidate reference gene with an M value ≤ 1.5 is considered an appropriate reference gene [3]. The M values for the six candidate reference genes we tested were all lower than 1.5 in both the MCAO mouse cortex and the OGD/R-injured N2a cells (Fig. 3). In the MCAO mouse cortex, HPRT and cyclophilin were the most stably expressed genes with M values of 0.394; in contrast, GAPDH exhibited the highest M value (0.611), which implied that it had the lowest expression stability among the six candidate reference genes (Fig. 3a). In the OGD/R-injured N2a cells, β-actin and cyclophilin possessed the lowest M values (both 0.618), which indicated that they had the best expression stabilities among the six selected candidate genes in the N2a cells that were subjected to OGD/R injury; in contrast, Sdha was the least stably expressed gene with the highest M value of the six genes (0.809) in the OGD/R-injured N2a cells (Fig. 3b). Overall, the results of GeNorm analysis were consistent with the Cp range results provided in Fig. 1.

Subsequently, the pairwise variation (V_n/n+1_) values determined by the GeNorm software were used to determine the optimal numbers of genes required for normalizing the RT-qPCR data in the ischemic models. The recommended cut-off threshold of V_n/n+1_ is 0.15 [16]. According to the results (Fig. 3c, d), V_3/4_ was less than 0.15 in the MCAO mouse cortex and V_4/5_ was < 0.15 in the OGD/R-injured N2a cells, which indicated three and four reference genes as the optimal numbers in the in vivo and in vitro models, respectively.

Next, NormFinder analysis was performed to evaluate the stabilities of the six candidate reference genes. NormFinder is a mathematical model of gene expression that estimates gene stability. This model is the only approach that enables the evaluation of not only the overall variation of the candidate genes but also the variations across the sample subgroups of the sample set [13]. Based on the results from NormFinder, the most stably expressed genes in the ischemic cortices of mice were 18S and HPRT. In the OGD/R-injured N2a cells, β-actin and cyclophilin were the most stable genes among the six candidate reference genes. These results were consistent with those from GeNorm (Table 2).

**Table 2:** Reference gene expression stability values in the tissues and cells based on several programs

Samples	Rank	geNorm		NormFinder		BestKeeper		ΔCp		General ranking
		Genes	M	Genes	Stability index	Genes	SD	Genes	Average SD	Genes
Tissues	1	HPRT	0.394	18S	0.193	HPRT	0.250	18S	0.568	HPRT
	2	Cyclophilin	0.394	HPRT	0.206	Cyclophilin	0.340	HPRT	0.572	18 s
	3	18S	0.512	Sdha	0.223	18S	0.370	Sdha	0.607	Cyclophilin
	4	Sdha	0.558	β-actin	0.223	β-actin	0.460	β-actin	0.623	Sdha
	5	β-actin	0.583	Cyclophilin	0.230	Sdha	0.520	Cyclophilin	0.626	actin
	6	GAPDH	0.611	GAPDH	0.247	GAPDH	0.550	GAPDH	0.662	GAPDH
Cells	1	β-actin	0.618	β-actin	0.228	18S	0.400	β-actin	0.671	β-actin
	2	Cyclophilin	0.618	Cyclophilin	0.310	Cyclophilin	0.410	Cyclophilin	0.734	Cyclophilin
	3	GAPDH	0.670	GAPDH	0.367	GAPDH	0.480	GAPDH	0.811	GAPDH
	4	18S	0.695	HPRT	0.419	β-actin	0.510	HPRT	0.84	18S
	5	HPRT	0.753	18S	0.439	HPRT	0.640	18S	0.877	HPRT
	6	Sdha	0.810	Sdha	0.451	Sdha	0.880	Sdha	0.921	Sdha

Subsequently, BestKeeper was used to rank the candidate reference genes by standard deviation (SD), i.e., a lower SD of the Cp values represented less variable expression compared with a higher SD value. In the BestKeeper analysis, genes with SDs > 1.0 are considered unstable and should be avoided, and all six reference genes we tested possessed SD values below 1.0 in both the MCAO mouse cortex and the OGD/R-injured N2a cells (Table 2), which indicates low expressional variation in the two models. BestKeeper ranked HPRT (SD: 0.25) and 18S (SD: 0.40) as the most stable reference genes in the MCAO mouse cortex and OGD/R-injured N2a cells, respectively.

**Fig. 4:**
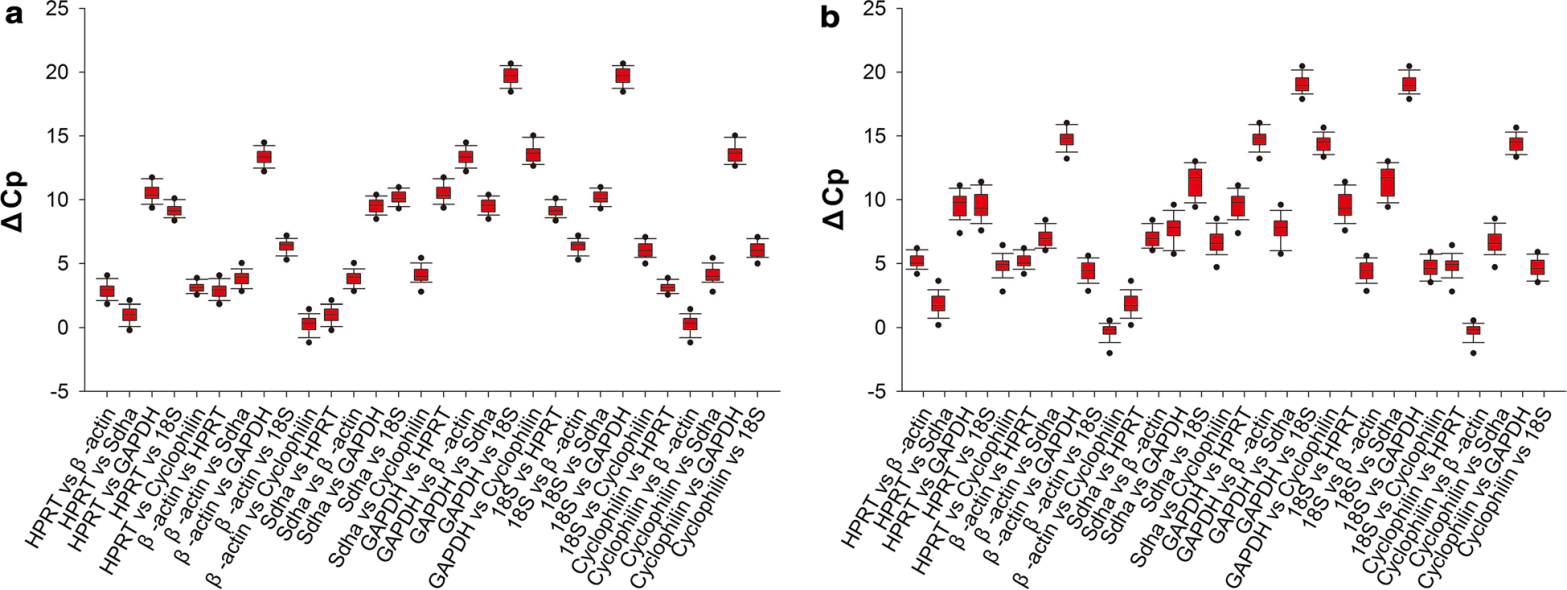
ΔCp method used in the selection of candidate reference genes. The ΔCp variability in the candidate reference genes is presented as the medians (lines), 25th percentiles to 75th percentiles (boxes), ranges (whiskers) and dots (5th and 95th percentiles) for samples of ischemic cerebral tissue from mice (**a**) and in OGD/R-injured N2a cells (**b**)

Among the six candidate reference genes, the comparative ΔCp method identified 18S and β-actin as the most stable genes in the cortices of the MCAO mice and the OGD/R-injured N2a cells, respectively, whereas GAPDH and Sdha were the least stable genes in the MCAO mouse cortex and the OGD/R-injured N2a cells, respectively (Table 3 and Fig. 4a, b).

**Table 3:** Candidate housekeeping gene comparisons

Sample	Mean ΔCp	SD	Mean SD	Mean ΔCp	SD	Mean SD
		Tissues			Cells	
18S versus HPRT	9.190	0.555	0.568	9.515	1.056	0.877
18S versus β-actin	6.340	0.518		4.329	0.767	
18S versus Sdha	10.180	0.519		11.342	1.143	
18S versus GAPDH	19.700	0.665		19.096	0.723	
18S versus cyclophilin	6.060	0.584		4.657	0.699	
HPRT versus β-actin	2.853	0.613	0.572	5.185	0.543	0.840
HPRT versus Sdha	0.990	0.623		1.827	0.839	
HPRT versus GAPDH	10.510	0.681		9.581	0.960	
HPRT versus 18S	9.192	0.555		9.515	1.056	
HPRT versus cyclophilin	3.130	0.389		4.858	0.802	
Sdha versus HPRT	0.990	0.623	0.607	1.827	0.839	0.921
Sdha versus β-actin	3.843	0.642		7.013	0.688	
Sdha versus GAPDH	9.521	0.569		7.754	1.010	
Sdha versus 18S	10.182	0.519		11.342	1.143	
Sdha versus cyclophilin	4.120	0.681		6.685	0.926	
β-actin versus HPRT	2.853	0.613	0.623	5.185	0.543	0.671
β-actin versus Sdha	3.843	0.642		7.013	0.688	
β-actin versus GAPDH	13.363	0.634		14.767	0.736	
β-actin versus 18S	6.339	0.518		4.329	0.767	
β-actin versus cyclophilin	0.277	0.711		0.327	0.618	
Cyclophilin versus HPRT	3.130	0.389	0.626	4.858	0.802	0.734
Cyclophilin versus β-actin	0.277	0.711		0.327	0.618	
Cyclophilin versus Sdha	4.120	0.681		6.685	0.926	
Cyclophilin versus GAPDH	13.640	0.763		14.439	0.624	
Cyclophilin versus 18S	6.062	0.584		4.657	0.699	
GAPDH versus HPRT	10.510	0.681	0.662	9.581	0.960	0.811
GAPDH versus β-actin	13.363	0.634		14.767	0.736	
GAPDH versus Sdha	9.521	0.569		7.754	1.010	
GAPDH versus 18S	19.702	0.665		19.096	0.723	
GAPDH versus cyclophilin	13.640	0.763		14.439	0.624

**Fig. 5:**
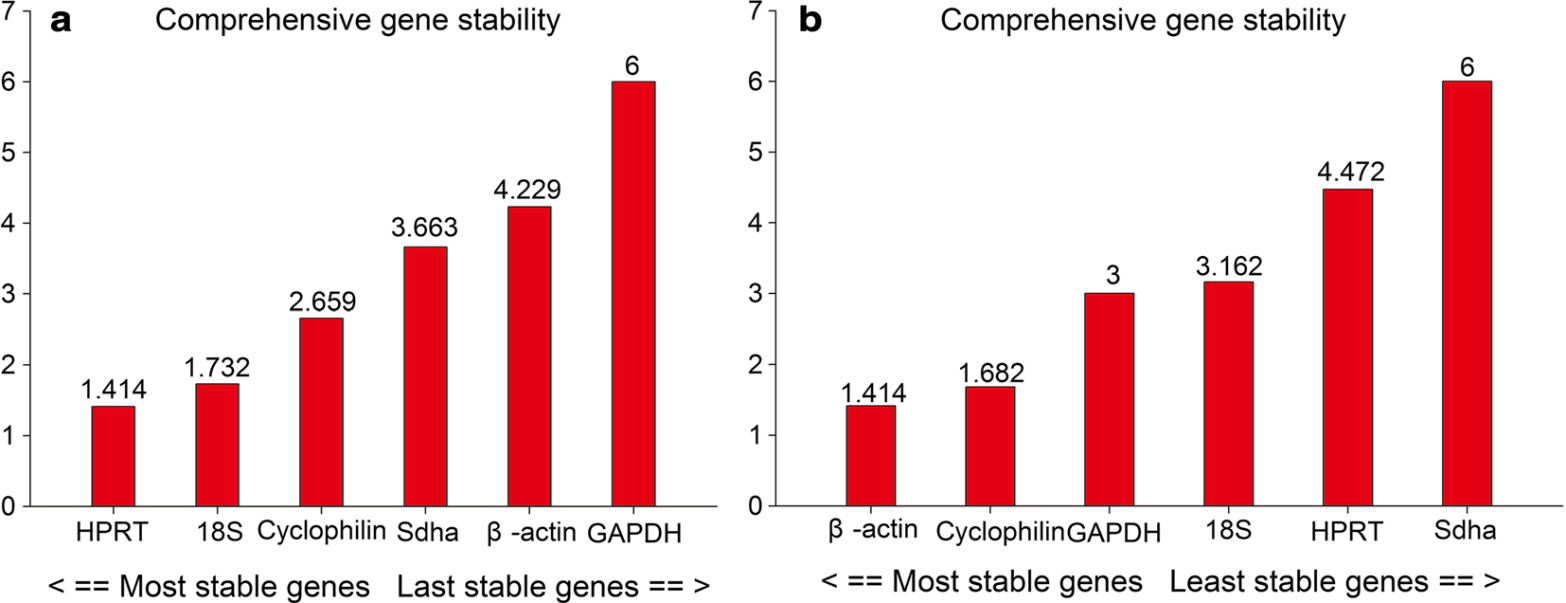
Comprehensive expression stabilities of the candidate reference genes analyzed by RefFinder in the ischemic cerebral tissue from mice (**a**) and in OGD/R-injured N2a cells (**b**)

Finally, RefFinder analysis, which combines several programs mentioned above and produces comprehensive outcomes, ranked the gene stabilities in the MCAO mouse cortex as HPRT > 18S > cyclophilin > Sdha > β-actin > GAPDH (Fig. 5a). In the OGD/R-injured N2a cells, gene stability decreased in the following order: β-actin > cyclophilin > GAPDH > 18S > HPRT > Sdha (Fig. 5b).

### Effects of reference genes on RT-qPCR data analysis

**Fig. 6:**
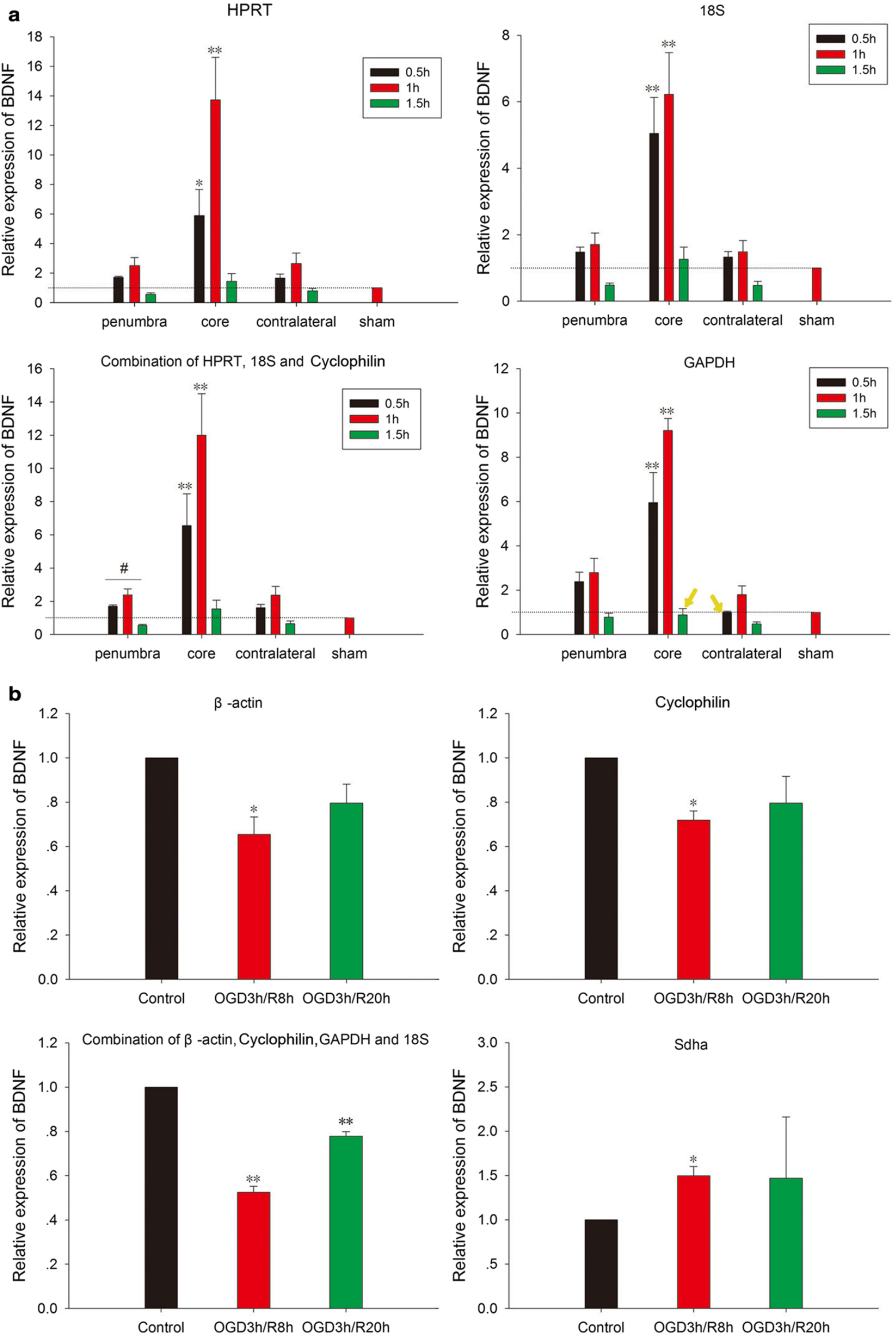
Relative expressions of the BDNF gene in the cortices of MCAO mice (**a**) and in OGD/R-injured N2a cells (**b**) using the reference genes or their best combinations. The bars indicate the means ± the SEMs. Asterisk Represents a significant difference at *P* < 0.05 compared with the sham. N = 3 per group

To determine the effect of reference genes on RT-qPCR analysis, we normalized brain-derived neurotrophic factor (BDNF) expression in the cortices of MCAO mice and in the OGD/R-injured N2a cells using HPRT, 18S, “the optimal combination” (i.e., HPRT, 18S and cyclophilin), or GAPDH. In the MCAO mouse cortex (Fig. 6a), the BDNF gene expression level was similar whether HPRT, 18S, “the optimal combination” (HPRT, 18S and cyclophilin), or GAPDH was used as the reference standard. BDNF gene expression began to increase after 0.5 h of MCAO followed by 24 h of reperfusion in the ischemic penumbra, the ischemic core and the contralateral area and then reached its expression peak after 1 h of MCAO followed by 24 h of reperfusion. After 1.5 h of MCAO followed by 24 h of reperfusion, the expression of BDNF became even lower than the sham group value in both the ischemic penumbra and contralateral area. At each time point of MCAO, BDNF exhibited the highest expression level in the ischemic core. Normalization of BDNF gene expression to GAPDH, which was the most unstable reference gene according to our analysis, produced results similar to those based on HPRT and 18S except when the ischemic core was subjected to 1.5 h of MCAO followed by 24 h of reperfusion.

In the OGD/R-injured N2a cells (Fig. 6b), the expression patterns of BDNF were similar when the data were normalized to either of the top two genes (β-actin or cyclophilin) or the optimal combination of genes (β-actin, cyclophilin, GAPDH and 18S). However, the normalization of BDNF gene expression to the most unstable reference gene, i.e., Sdha, produced markedly different results. Therefore, the selection of a reference gene influences the results of RT-qPCR analyses. In other words, reliable results depend on the choice of suitable reference genes for normalization.

## Discussion

In the present study, we evaluated the stability of six candidate reference genes (HPRT, β-actin, Sdha, GAPDH, 18S and cyclophilin) in two ischemia models: N2a cells at several time points after OGD/R injury and the intraluminal filament model of MCAO in C57 mice.

The two most stable genes (HPRT, 18S/cyclophilin) in the in vivo model were ranked differently depending on the program used to analyze the data (geNorm, NormFinder, BestKeeper, ΔCp method and RefFinder). In the in vitro model of N2a cells, β-actin and cyclophilin/GAPDH were ranked as the two most stable genes. In contrast, GAPDH and Sdha were the most variable genes in the in vivo and in vitro models, respectively.

GAPDH is a glycolytic enzyme usually that is used as a housekeeping gene in RT-qPCR analysis. However, whether GAPDH is suitable for RT-qPCR normalization in MCAO models is controversial [17]. There have been five studies that have reported on the suitability of reference genes for the permanent MCAO model, and all five studies have evaluated the expression stability of GAPDH. Among the results, three studies recommended GAPDH as a suitable control, whereas the other two regarded GAPDH as an unsuitable control, which is more consistent with our results; however, the in vivo model we used was different from the model used in these reports (transient MCAO in mice vs permanent MCAO in rats). Regarding the mechanism, an increasing amount of research demonstrates that GAPDH tends to translocate into the nuclei of neurons in several neurodegenerative disorders, such as Huntington disease [18], Alzheimer’s disease [19] and stroke [20]. Specifically, GAPDH has been indicated to be a proapoptotic protein and to be dynamically expressed in the rat brain over the course of ischemia/reperfusion damage [20], which revealed the inapplicability of GAPDH for RT-qPCR data normalization in rat brains. We speculate that GAPDH underwent the same biological process in transiently ischemic mouse brains. However, there is a lack of studies investigating the applicability of GAPDH for RT-qPCR data normalization in OGD/R-injured N2a cells, and the present study indicated that GAPDH was a suitable control in OGD/R-injured N2a cells.

BDNF is a key neuroprotective protein in the brain and promotes neuronal survival and differentiation [21]. The literature has documented the association of BDNF with many serious diseases, such as Alzheimer’s disease [22], depression [23], and stroke [24]. In our research, we validated the BDNF expression by comparing the utility of HPRT, 18S, the optimal combination and GAPDH as reference genes in RT-qPCR data analysis. This comparison demonstrated that the BDNF gene expression trends were similar when normalized by either of the top two candidate reference genes or the optimal combination; the expression of BDNF mRNA was increased in the ischemic penumbra, the ischemic core and even the contralateral area after 0.5 and 1 h of MCAO followed by 24 h of reperfusion and was decreased after 1.5 h of MCAO followed by 24 h of reperfusion. Then, BDNF expression was reduced to a level lower than that in the sham samples after 1.5 h of MCAO followed by 24 h of reperfusion. Although GAPDH was the most unstable gene in the MCAO mice, several parameters calculated with evaluation software revealed that GAPDH was still suitable for normalization in RT-qPCR analysis. For this reason, only a very small difference in GAPDH was observed compared with the other three MCAO groups.

The candidate reference genes were selected based on a literature search to identify genes that are commonly used for normalization in ischemic stroke models. However, other potentially suitable housekeeping genes were not selected. Because the scope was thus limited, we examined relatively few candidate reference genes. Despite these limitations, we believe that the evidence in this work provides helpful information for gene expression studies.

## Conclusions

The present study indicates that a combination of HPRT, 18S, and cyclophilin is the most suitable set of reference genes for analyses of mice subjected to ischemic stroke, while the combination of β-actin, cyclophilin, GAPDH and 18S is recommended as the most suitable set of reference genes for analyses of OGD/R-injured N2a cells.

## Methods

### Cell culture and OGD/R injury

The N2a cell line was provided in 2016 by the Center for Drug Research and Development of Zhujiang Hospital, Southern Medical University. The N2a cells were cultured in high-glucose Dulbecco’s modified Eagle medium (DMEM) with 10% fetal bovine serum (FBS) in a humidified atmosphere of 5% CO_2_ at 37 °C. To model OGD/R injury, we washed the cells three times with phosphate-buffered saline (PBS) and incubated them in glucose-free DMEM. The cells were then subjected to treatment in a sealed anaerobic chamber with a humidified gas mixture of 95% N_2_ and 5% CO_2_ at 37 °C for 3 h. After OGD exposure, the cells were maintained under normal culture conditions for 0, 4, 8, 12, 16 or 20 h for reoxygenation. The control cells were cultured in normal culture media and under normal conditions for the whole duration of the experiment. Cell viability was measured using a cell counting kit-8 (CCK-8).

### Animals

Male C57BL/6 J mice (7–8 weeks of age, 18–21 g in weight) were purchased from Guangdong Medical Laboratory Animal Center. The mice were housed in cages maintained in a regulated environment (12-h:12-h light/dark cycle) and were supplied with water and food ad libitum. All animal procedures used in the experiments were approved by the local Animal Use and Care Committee of Guangdong Medical Laboratory Animal Center (approval ID: B201608-6). All institutional guidelines for animal welfare and experimental conduct were followed. All efforts were made to minimize suffering.

### Focal ischemia and reperfusion

Intraluminal middle cerebral artery occlusion (MCAO) was induced in the mice by the intraluminal suture method. Briefly, the animals were anesthetized before surgery with isoflurane (3% initially, 1–1.5% for maintenance) in O_2_ and N_2_O (1:3), and their body temperature was maintained at 36.8 ± 0.2 °C with a heating blanket throughout the procedure until the mice awoke from anesthesia. After the right common carotid artery (CCA), the right external carotid artery (ECA) and the right internal carotid artery (ICA) were isolated, 6–0 sutures were tied at the origin of the ECA and at the distal end of the ECA. A silicon-coated filament (0.21 ± 0.02 mm in diameter) was introduced into the ECA until it reached the CCA. Then, the direction of the filament was changed so that it entered the ICA and finally lodged in the anterior cerebral artery (ACA) to block the blood flow into the middle cerebral artery (MCA). After several hours of occlusion (0.5, 1 or 1.5 h), the filament was withdrawn for 24 h of reperfusion. Sham-operated mice underwent the same procedures with the exception of the filament insertion. After 24 h of reperfusion, the animals were sacrificed under anesthesia, and the brains were collected on iced plates as quickly as possible for further study. The infarction was detected using 2% 2, 3, 5-triphenyltetrazolium chloride (TTC)-stained specimens from four coronal sections per mouse. The infarct volume was calculated according to the following formula:

Infarct volume = (total volume of the ipsilateral hemisphere − the volume of normal tissue in the ipsilateral hemisphere)/total volume of the ipsilateral hemisphere × 100%.

### Preparation of samples and RNA extraction

The ischemic core, penumbra and the non-ischemic cortex were separated as experimental samples. Briefly, the brain was sectioned into three parts, and the second part (between 2 and 6 mm from the anterior tip of the frontal lobe for a total of 4 mm thickness) was dissected for cortical samples from the ischemic core and penumbra. First, the midline between the two hemispheres was identified, and a longitudinal cut approximately 1 mm from the midline through the ipsilateral hemisphere was made to avoid the mesial hemispheric structures in which the blood is supplied primarily by the anterior cerebral artery. Then, the ischemic core and penumbral cortex were isolated from the remainder of the ipsilateral tissue. The non-ischemic cortex was collected from the second section of the corresponding contralateral portion.

Total RNA was extracted from the tissue or N2a cells using TRIzol^®^ LS (Invitrogen, Stockholm, Sweden) according to the manufacturer’s instructions. The quantity and quality of the RNA were measured with a NanoDrop ND-2000. The total amounts of RNA obtained did not differ significantly between the samples.

### Real-time reverse transcription quantitative polymerase chain reaction (RT-qPCR)

For cDNA synthesis, 2.5 ng of total RNA was reverse transcribed (RT) into cDNA using the PrimeScript™ RT Reagent Kit with gDNA Eraser (Perfect Real Time, TaKaRa, Japan) according to the manufacturer’s instructions. The real-time monitoring of PCR amplification reaction was performed on a Real-Time PCR Detection System (Light Cycler 480, Roche) using SYBR^®^ Premix Ex Taq™ (Tli RNaseH Plus, TaKaRa, Japan) according to the manufacturer’s instructions. The real-time PCRs were conducted at 95 °C for 5 min, followed by 40 cycles of 15 s at 94, 60 °C for 15 s, and 72 °C for 30 s in sequence. The primers used for RT-qPCR are listed in Additional file 1: Table S1. The fold change in the expression of each gene was calculated using the ΔCt method.

### Statistical analysis

SPSS 16.0 software was used to analyze the mRNA expression of the target gene in all experimental groups using one-way ANOVA with the significance level set at 0.05.

## Additional files

**Additional file 1: Table S1.** PCR primers targeting the reference genes and the target gene BDNF.

**Additional file 2: Figure S1.** The specificities of the primers used in the RT-qPCR analysis. A. Dissociation curves with single peaks generated from the reaction. B. Electrophoresis on 2% agarose gel of the RT-qPCR products of reference genes.

**Additional file 3: Figure S2.** The establishment of the in vivo and in vitro models of ischemic stroke. A. Representative images showing TTC-stained brain sections. B. The quantitative analysis of brain infarct volumes in the mice. C. The cell viabilities of the OGD/R-injured N2a cells were measured using the CCK-8 assay. The data represent the means ± the SEMs of three independent experiments. *P < 0.05 and **P < 0.01 versus the sham or control.

## Abbreviations

RT-qPCR: Real-time reverse transcription quantitative polymerase chain reaction
MCAO: middle cerebral artery occlusion
OGD/R: oxygen–glucose deprivation/reoxygenation
GAPDH: glyceraldehyde-3-phosphate dehydrogenase
HPRT: hypoxanthine phosphoribosyltransferase
Sdha: succinate dehydrogenase complex, subunit A
BDNF: brain-derived neurotrophic factor
