# Supplementary material for: Evaluation of reference genes for gene expression studies in mouse and N2a cell ischemic stroke models using quantitative real-time PCR
Source: BMC Neurosci. 2018 Feb 1;19:3. doi: 10.1186/s12868-018-0403-6 (PMC5795833; doi:10.1186/s12868-018-0403-6)
Supplement: Supplementary file 1 — Additional file 1: Table S1. PCR primers targeting the reference genes and the target gene BDNF. [file 12868_2018_403_MOESM1_ESM.pdf]

Table S1. PCR primers targeting the reference genes and the target gene BDNF.

Gene symbol	Accession number	Primer sequence (5'-3')	Product length (bp)
HPRT	NM_013556	AGTGTTGGATACAGGCCAGAC<br>CGTGATTCAAATCCCTGAAGT	103
18S	NR_003278	CCTGGATACCGCAGCTAGGA<br>GCGGCGCAATACGAATGCCCC	111
β-actin	NM_007393	GTACCACCATGTACCCAGGC<br>AACGCAGCTCAGTAACAGTCC	247
Sdha	NM_023281	ACACAGACCTGGTGGAGACC<br>GCACAGTCAGCCTCATTCAA	289
GAPDH	NM_008084	AGCTACTCGCGGCTTTACG<br>ATCCGTTCACACCGACCTTC	246
Cyclophilin	NM_008907	TCCGACTGTGGACAGCTCTA<br>ATTGCGAGCAGATGGGGTAG	105
BDNF	NM_007540	GGGAAATCTCCTGAGCCGAG<br>AACTCTCATCCACCTTGGCG	363
