# Supplementary material for: Evaluation of reference genes for gene expression studies in mouse and N2a cell ischemic stroke models using quantitative real-time PCR
Source: BMC Neurosci. 2018 Feb 1;19:3. doi: 10.1186/s12868-018-0403-6 (PMC5795833; doi:10.1186/s12868-018-0403-6)
Supplement: Supplementary file 2 — Additional file 2: Figure S1. The specificities of the primers used in the RT-qPCR analysis. A. Dissociation curves with single peaks generated from the reaction. B. Electrophoresis on 2% agarose gel of the RT-qPCR products of reference genes. [file 12868_2018_403_MOESM2_ESM.pdf]

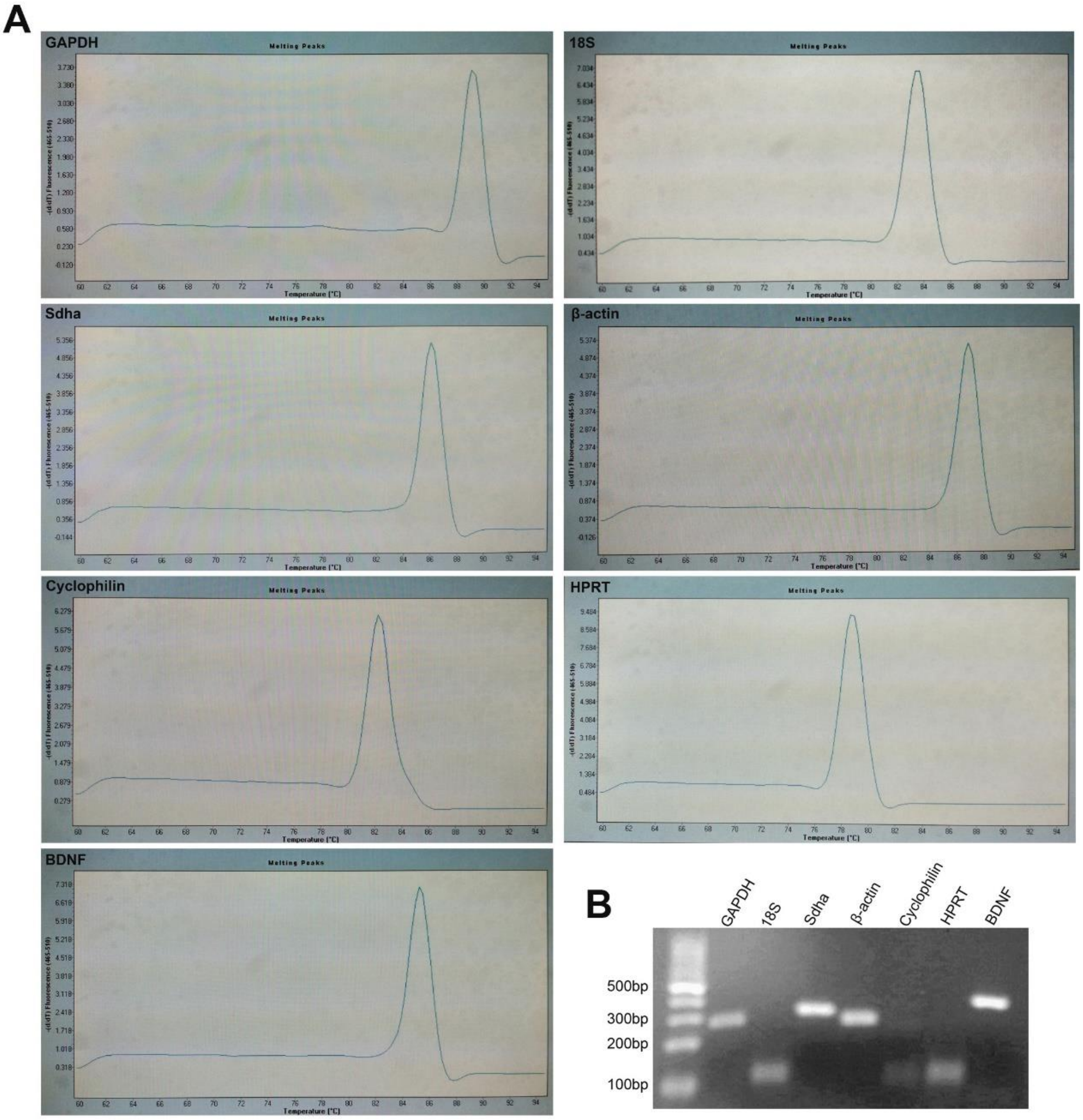

Figure S1. The specificities of the primers used in the RT-qPCR analysis. A. Dissociation curves with single peaks generated from the reaction. B. Electrophoresis on 2% agarose gel of the RT-qPCR products of reference genes.
