# Supplementary material for: Evaluation of reference genes for gene expression studies in mouse and N2a cell ischemic stroke models using quantitative real-time PCR
Source: BMC Neurosci. 2018 Feb 1;19:3. doi: 10.1186/s12868-018-0403-6 (PMC5795833; doi:10.1186/s12868-018-0403-6)
Supplement: Supplementary file 3 — Additional file 3: Figure S2. The establishment of the in vivo and in vitro models of ischemic stroke. A. Representative images showing TTC-stained brain sections. B. The quantitative analysis of brain infarct volumes in the mice. C. The cell viabilities of the OGD/R-injured N2a cells were measured using the CCK-8 assay. The data represent the means ± the SEMs of three independent experiments. *P < 0.05 and **P < 0.01 versus the sham or control. [file 12868_2018_403_MOESM3_ESM.pdf]

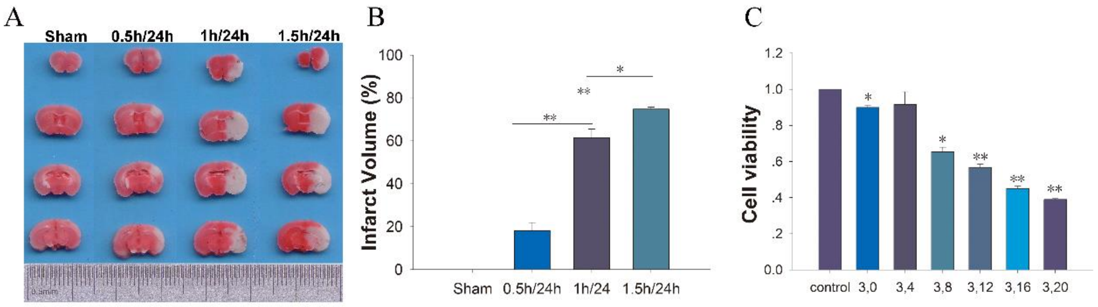

Figure S2. The establishment of the in vivo and in vitro models of ischemic stroke. A. Representative images showing TTC-stained brain sections. B. The quantitative analysis of brain infarct volumes in the mice. C. The cell viabilities of the OGD/R-injured N2a cells were measured using the CCK-8 assay. The data represent the means ± the SEMs of three independent experiments. *P < 0.05 and **P < 0.01 versus the sham or control.
